# Fluid management among outpatients with chronic heart failure: a cross-sectional study

**DOI:** 10.3389/fcvm.2026.1634045

**Published:** 2026-02-16

**Authors:** Yan Wu, Yan Qian, Lingyan Zhu

**Affiliations:** 1 Department of Joint Surgery, Shanghai Sixth People’s Hospital, Shanghai, China; 2 Department of Nursing, Shanghai Sixth People’s Hospital, Shanghai, China

**Keywords:** chronic heart failure, fluid management, influencing factors, multiple linear regression analysis, self-efficacy, social support

## Abstract

**Introduction:**

Chronic heart failure (CHF) significantly impairs patients’ quality of life and poses a substantial clinical burden. Fluid management is a critical aspect of self-care in CHF, particularly in out-of-hospital settings. However, the current status and factors influencing fluid management abilities among CHF patients outside the hospital remain insufficiently explored. This study aimed to examine the current state of out-of-hospital fluid management in CHF patients and identify factors influencing their self-management behavior.

**Methods:**

A total of 184 patients were included in this cross-sectional study. Participants completed questionnaires, including a general information questionnaire, the Heart Failure Weight Management Questionnaire, the General Self-Efficacy Scale (GSES), the Social Support Rating Scale (SSRS), and the Generalized Anxiety Disorder-7 scale (GAD-7). Data were analyzed using multiple linear regression to identify predictors of out-of-hospital fluid management ability.

**Results:**

The mean total score for out-of-hospital fluid management was 28.7 ± 5.4, with the highest subscore observed in weight monitoring (8.1 ± 2.1). Multiple linear regression revealed that younger age, higher educational level, lower anxiety score, fewer comorbidities, lower BMI, stronger social support, and greater self-efficacy were significantly associated with better fluid management performance (*P* < 0.05).

**Conclusion:**

The findings indicate that fluid management ability in CHF patients is influenced by a combination of physiological, psychological, and social factors. Tailored interventions addressing these aspects are necessary to enhance self-care competence and improve prognosis in CHF patients living outside the hospital.

## 1 Introduction

Chronic heart failure (CHF) is a specific form of heart failure that occurs when the heart is unable to pump blood efficiently to meet the body's metabolic needs, leading to symptoms such as fatigue, fluid retention, and shortness of breath. CHF is a progressive condition, typically defined as the inability of the heart to maintain adequate circulation despite increased filling pressures. It severely impairs patients' quality of life and imposes substantial healthcare and socioeconomic burdens due to high hospitalization rates and mortality (1, 2). With the aging population, increasing cardiovascular risk factors, and improved survival of patients with heart failure due to advances in medical care, the prevalence of CHF continues to rise in both developed and developing countries (3–5).

Effective fluid management is a cornerstone of CHF care, as maintaining optimal fluid balance through appropriate control of fluid intake and output is critical to symptom relief and disease stabilization. Impaired cardiac function can lead to fluid retention, increasing cardiac workload and predisposing patients to pulmonary or peripheral edema and other complications (6, 7). Therefore, proper fluid management not only alleviates symptoms but also improves prognosis and reduces hospital readmissions.

According to the 2023 European Society of Cardiology (ESC) and 2022 ACC/AHA/HFSA heart failure guidelines, patient education and self-care behaviors—including weight monitoring, daily fluid and sodium restriction, and early recognition of symptom changes-are essential components of long-term management (8, 9). However, effective out-of-hospital fluid management remains challenging, as it requires patients to continuously monitor their fluid intake, weight, and symptoms, and to adjust behaviors based on medical advice. These tasks demand adequate health literacy, self-management skills, and family or social support (10, 11). Most existing studies have focused on fluid management practices in hospital settings, whereas limited research has examined the current status and determinants of out-of-hospital fluid management among CHF patients (12–14). Understanding these factors is vital for designing targeted interventions to enhance self-care abilities and improve clinical outcomes.

Therefore, this study aimed to examine the current status of out-of-hospital fluid management among CHF patients and identify the factors influencing self-management behaviors, providing evidence to guide individualized educational and behavioral strategies to optimize fluid control and quality of life.

## 2 Materials and methods

### 2.1 Study participants

This study employed a convenience sampling method, which was chosen for this initial, single-center investigation due to its operational feasibility. It allowed for the efficient identification and enrollment of eligible patients from the available clinical population. Participants were recruited from the outpatient department of our hospital during their scheduled follow-up visits between October 2019 and December 2022. A trained research nurse approached potential participants in the outpatient waiting area after their clinical consultation. The study's purpose, procedures, and voluntary nature were explained. Patients who met the inclusion criteria and provided written informed consent were subsequently enrolled. Inclusion criteria were: (1) diagnosis of CHF according to the 2014 New York Heart Association (NYHA) criteria; (2) NYHA functional class II–IV; (3) age ≤70 years; and (4) provision of written informed consent. Exclusion criteria: patients with acute myocardial infarction; the presence of other serious comorbidities, such as malignancy or severe renal impairment [typically defined as an estimated glomerular filtration rate (eGFR) < 30 mL/min/1.73 m²] (9); Patients with mental disorders or unconsciousness. The sample size was calculated based on the formula for estimating a population proportion in a cross-sectional study: *n* = [Zα/2]² × P(1-P)/δ², where *n* is the required sample size, Zα/2 = 1.96 (for α = 0.05), P is the assumed proportion, and *δ* is the allowable error. To ensure an adequately sized sample for this descriptive study, a conservative approach was used: P was set to 0.5 (maximizing the required *n*), and δ was set to 0.075 (15% of P). A minimum sample size of 171 was calculated. Considering an estimated 10% attrition rate, the final target sample size was set at 189.

### 2.2 Measures and instruments

#### 2.2.1 General information questionnaire

A self-designed questionnaire was used to gather basic information and health-related data of CHF patients. The questionnaire included the following aspects: (1) demographic characteristics, including age, gender, marital status, education level, monthly family income per capita, occupational status, payment method of medical expenses and place of residence. (2) Health behavior: the history of smoking and drinking was recorded in detail. (3) disease-related information, containing the patient's course of CHF and family history. (4) Psychological status: the patient's psychological characteristics (such as introversion, extroversion, and neutrality) were evaluated. (5) Comorbidities: whether the patient had other comorbidities was collected. (6) Clinical indicators: body mass index (BMI), left ventricular ejection fraction (LVEF) and pro-brain natriuretic peptide (Pro-BNP) were recorded by medical staff.

#### 2.2.2 Heart failure weight management questionnaire

The Weight Management in Heart Failure Questionnaire is a professional tool used to assess out-of-hospital fluid management ability in CHF patients (15). The questionnaire was developed by Wang et al. It contains 16 items and is divided into four dimensions: weight monitoring, knowledge related to weight management, attitude, and behavior management. Questionnaires are scored using Likert scales, with each item scored from 0 to 2 or 0 to 3, and total scores range from 0 to 42, with lower scores indicating poorer weight-management skills. The questionnaire has good reliability and validity through scientific verification. The content validity was 0.88, the Cronbach's *α* coefficient of the total questionnaire was 0.843, and the test-retest reliability was 0.833.

#### 2.2.3 Social support rating scale (SSRS)

The SSRS included three main dimensions: subjective support (four items), objective support (three items), and the utilization of support (three items) (16). For items 1 to 4 and 8 to 10, only one item was selected. For items 1, 2, 3 and 4, scores were recorded as 1, 2, 3 and 4 respectively. The fifth item was divided into 5 items A, B, C, D, E, and each item was scored 1–4 points from “none” to “full support”, that is, “none” scored 1 point, “very little” scored 2 points, “general” scored 3 points, and “full support” scored 4. Article 6, 7 if you answer, “No source”, you will be scored 0. Those who answered, “the following sources”, counted several sources. The total score on the scale had a range of 12 to 66, its lower scores reflecting lower levels of social support received by patients. <20 points mean social support is less, 20–30 points denotes social support is general, ≥30 points mean social support is satisfactory. The internal consistency coefficient of the scale had a scope of 0.890 to 0.940, the test-retest reliability was 0.920.

#### 2.2.4 General self-efficacy scale (GSES)

The GSES contains ten items, each item is scored using a Likert 4-point scale, and participants choose the option that best fits their own feelings and experiences for each item (17). The GSES's total score ranged from 10 to 40, with higher total scores indicating greater self-efficacy of the individual. Low self-efficacy was defined as total score < 25, high self-efficacy meant total score > 35, and medium self-efficacy was total score ≤35. This scale' Cronbach's *α* coefficient was 0.853, manifesting that it had good reliability.

#### 2.2.5 GAD-7 scale

Generalized Anxiety Disorder-7 (GAD-7) is a self-rate scale developed by Spitzer et al. for the assessment of generalized anxiety symptoms in individuals (18). The scale involves seven items, each is scored by a Likert 4-point method (3 = almost every day, 2 = more than half the time, 1 = some days, 0 = not at all), and participants rate the frequency of symptoms during the previous 2 weeks. GAD-7's total scores were in the range of 0 to 21, and lower scores indicating less severe anxiety symptoms. According to the total score, the anxiety level can be divided into 0–4 as not anxious, 5–9 as mildly anxious, 10–14 as moderately anxious, and 15–21 as severely anxious. The Cronbach's α coefficient of the scale was 0.92, indicating that it had good internal consistency and reliability.

#### 2.2.6 PHQ-9 scale

Patient Health Questionnaire-9 (PHQ-9) was utilized to assess the severity of depressive symptoms in individuals, which is a self-rating scale developed by Kroenke et al. The PHQ-9 consists of 9 items, each on a four-point Likert scale (3 = almost every day, 2 = more than half the time, 1 = some days, 0 = not at all), in which participants rate the frequency of symptoms during the previous 2 weeks (19). The PHQ-9' total score scopes from 0 to 27, and lower scores pointing to less severe depressive symptoms. The degree of depression was divided into 0–4 as not depressed, 5–9 as depressed mildly, 10–14 as depressed moderately, 15–19 as moderately to severely depressed, and 20–27 as depressed severely. The PHQ-9's Cronbach's α coefficient was 0.89, manifesting that it had good validity and reliability.

### 2.3 Data collection procedure

Before the formal investigation, all the investigators accepted detailed training, covering the core concepts of the questionnaire, the filling specifications and the unified instructions. Investigators need to pass the assessment to ensure that they understand and master the survey process before they can formally participate in the survey. All surveys were conducted face-to-face, and the investigator briefed the patients in detail on the purpose and importance of the survey and instructed the patients on how to correctly fill out the questionnaire. Investigators will provide immediate and clear explanations for patients' questions during the filling process. Each questionnaire was reviewed in detail by the investigator upon completion to ensure completeness and accuracy. Due to this rigorous, face-to-face data collection process with immediate clarification of any missing items, all distributed questionnaires were fully completed and retained for analysis. In this research, 189 questionnaires were distributed and all 189 were collected. After detailed verification, 5 questionnaires were excluded due to response patterns or inconsistencies, resulting in 184 valid questionnaires for analysis.

### 2.4 Statistical analysis

Data were analyzed using SPSS 22.0 (IBM Corp., Armonk, NY, USA). Continuous variables with normal distribution were presented as mean ± standard deviation (SD), while those with non-normal distribution were described using medians and interquartile ranges. Categorical data were expressed as frequencies and percentages. For the Likert-scale questionnaires-specifically the Heart Failure Weight Management Questionnaire (HFWMQ), General Self-Efficacy Scale (GSES), Social Support Rating Scale (SSRS), Generalized Anxiety Disorder-7 (GAD-7), and Patient Health Questionnaire-9 (PHQ-9)-the total scores were treated as continuous variables in the multiple linear regression analysis. In the univariate analysis, the scores of GAD-7, PHQ-9, SSRS, and GSES were categorized into groups based on their standard, validated cut-off points to facilitate group comparisons using one-way analysis of variance (ANOVA) or t-test. We used multiple linear regression analysis to identify the independent factors associated with fluid management ability. Prior to regression, model assumptions (linearity, normality of residuals, homoscedasticity, and independence) were examined. Residual plots and Durbin-Watson statistics were used to assess independence, while Q-Q plots and standardized residual distributions were used to evaluate normality. These diagnostic checks confirmed that the model assumptions were reasonably met. All statistical tests were two-sided, and a *P*-value < 0.05 was considered statistically significant.

## 3 Results

### 3.1 Participant characteristics and fluid management scores

A total of 184 patients with chronic heart failure were included in the final analysis. The demographic and clinical characteristics of the participants are summarized in Table 2. The overall mean score for out-of-hospital fluid management, as measured by the Heart Failure Weight Management Questionnaire, was 28.7 ± 5.4, indicating a moderate level of self-management ability. As detailed in Table 1, the dimension of weight monitoring received the highest mean score (8.1 ± 2.1), while behavior management scored the lowest (6.8 ± 1.7).

**Table 1:** Out-of-hospital liquid management in patients with chronic heart failure (*n* = 184, x¯ ± s).

Dimensions	Total points
Weight monitoring	8.1 ± 2.1
Knowledge related to weight management	6.5 ± 1.9
Belief	7.3 ± 2.0
Behavior management	6.8 ± 1.7

**Table 2:** Single factor analysis of the current situation of liquid management in patients with chronic heart failure outside hospital (*n* = 184, x¯ ± s).

Items	*N* (%)	Score of heart failure weight management questionnaire	t/F	*P*
Gender			−1.278	0.203
Male	100 (54.3)	28.2 ± 5.3		
Female	84 (45.7)	29.1 ± 5.4		
Age (years old)			6.874	0.001
<50	50 (27.2)	26.3 ± 4.8		
50–65	90 (48.9)	29.7 ± 5.0		
>65	44 (23.9)	31.6 ± 5.3		
Educational Level			5.234	0.004
Junior school and below	64 (34.8)	26.8 ± 5.0		
High school or technical secondary school	70 (38.0)	28.9 ± 5.1		
Junior college or above	50 (27.2)	31.2 ± 5.4		
Marital status			1.142	0.289
Married	130 (70.7)	29.0 ± 5.5		
Unmarried	30 (16.3)	27.7 ± 5.2		
Divorced or widowed	24 (13.0)	28.1 ± 5.4		
Monthly household income per capita (Yuan)			2.132	0.123
<5,000	80 (43.5)	27.0 ± 5.1		
5,000–10,000	70 (38.0)	29.4 ± 5.3		
>10,000	34 (18.5)	30.9 ± 5.5		
Status of occupation			−0.672	0.502
At the office	100 (54.3)	28.4 ± 5.2		
Resigned or retired	84 (45.7)	28.9 ± 5.3		
Payment of medical expenses			1.234	0.295
Self-paying	60 (32.6)	29.2 ± 5.3		
Medical insurance	104 (56.5)	28.1 ± 5.2		
Else	20 (10.9)	28.7 ± 5.4		
Place of residence			0.892	0.373
Cities	110 (59.8)	28.8 ± 5.4		
Rural areas	74 (40.2)	28.1 ± 5.2		
Smoking history			1.924	0.056
Yes	110 (59.8)	27.8 ± 5.1		
No	74 (40.2)	29.2 ± 5.4		
Drinking history			1.187	0.237
Yes	95 (51.6)	28.5 ± 5.3		
No	89 (48.4)	28.7 ± 5.4		
Course of disease(year)			1.732	0.085
<1	60 (32.6)	27.1 ± 5.1		
1–5	90 (48.9)	28.9 ± 5.3		
>5	34 (18.5)	30.3 ± 5.5		
Family history			0.984	0.327
Yes	100 (54.3)	28.7 ± 5.4		
No	84 (45.7)	28.4 ± 5.2		
Mental state			1.783	0.067
Introverted	60 (32.6)	30.0 ± 5.3		
Extroverted	90 (48.9)	27.5 ± 5.1		
Neutral	34 (18.5)	28.8 ± 5.2		
Complication			4.932	0.008
Yes	120 (65.2)	30.3 ± 5.5		
No	64 (34.8)	27.6 ± 5.0		
Body mass index (BMI)			3.678	0.012
≤23.9	74 (40.2)	27.4 ± 5.1		
24.0–27.9	80 (43.5)	29.2 ± 5.2		
>27.9	30 (16.3)	30.8 ± 5.3		
Pro-brain natriuretic peptide (Pro-BNP)			1.382	0.169
Normal	50 (27.2)	29.5 ± 5.4		
Elevated	134 (72.8)	28.2 ± 5.2		
LVEF			2.019	0.056
≥50%	70 (38.0)	29.8 ± 5.4		
<50%	114 (62.0)	28.0 ± 5.3		
Anxiety score (GAD-7)			5.112	0.003
No anxiety	70 (38.0)	27.5 ± 5.0		
Mild anxiety	60 (32.6)	29.1 ± 5.1		
Moderate anxiety	40 (21.7)	31.3 ± 5.3		
Severe anxiety	14 (7.6)	32.5 ± 5.4		
Depression score (PHQ-9)			1.876	0.062
Not depressed	84 (45.7)	27.5 ± 5.1		
Mild depression	60 (32.6)	28.8 ± 5.2		
Moderate depression	30 (16.3)	30.5 ± 5.3		
Moderate to severe depression	10 (5.4)	31.9 ± 5.5		
Social support rating scale (SSRS)			6.219	<0.001
Low	64 (34.8)	31.2 ± 5.4		
Middle	90 (48.9)	28.3 ± 5.1		
High	30 (16.3)	26.9 ± 5.0		
General self-efficacy scale (GSES)			6.045	<0.001
Low	70 (38.0)	31.4 ± 5.3		
Middle	80 (43.5)	28.8 ± 5.1		
High	34 (18.5)	26.7 ± 5.0		

### 3.2 Univariate analysis of current situation of out-of-hospital fluid management in CHF patients

Univariate analysis (Table 2) revealed that several factors were significantly associated (*P* < 0.05) with the total fluid management score. These included age, educational level, the presence of comorbidities, BMI, anxiety level (GAD-7 score), social support level (SSRS score), and self-efficacy level (GSES score). Other variables, such as gender, marital status, income, and clinical indicators like LVEF and Pro-BNP, showed no significant association.

### 3.3 Multiple linear regression analysis of affecting elements of out-of-hospital fluid management ability in CHF patients

The variable assignment methods used for regression modeling are summarized in Table 3. The results of the multiple linear regression analysis are presented in Table 4. The model was statistically significant (*F* = 49.732, *P* < 0.001) and explained approximately 66.8% of the variance in fluid management scores (Adjusted *R*^2^ = 0.668). Independent factors significantly associated with better fluid management ability were: higher educational level, stronger social support, and greater self-efficacy. Factors significantly associated with poorer fluid management ability were: older age, the presence of comorbidities, higher BMI, and higher levels of anxiety. The detailed regression coefficients, confidence intervals, and *P*-values for each variable are provided in Table 4.

**Table 3:** Method of assignment.

Variables	Method of assignment
Age (years old)	“<50” = 00(reference group), “50–65” = 01, “>65” = 10
Educational level	“Junior school and below” = 00(reference group), “High school or technical secondary school” = 01, “Junior college or above” = 10
Comorbidities	“No” = 0(reference group), “Yes” = 1
Body mass index (BMI)	“≤23.9” = 00(reference group), “24.0–27.9” = 01, “>27.9” = 10
Anxiety score (GAD-7)	“No anxiety” = 00 (reference group), “Mild anxiety” = 01, “Moderate anxiety” = 10, “Severe anxiety” = 11
Social support rating scale (SSRS)	“Low” = 00(reference group), “middle” = 01, “high” = 10
General self-efficacy scale (GSES)	“Low” = 00(reference group), “middle” = 01, “high” = 10

**Table 4:** Multiple linear regression analysis of affecting factors of out-of-hospital fluid management ability in CHF patients (*n* = 184).

Items	Regression coefficient	Standard error	Standard regression coefficient	t	*P*	95% CI
Constant	27.183	1.056	–	25.749	<0.001	25.094–29.272
Age (with <50 years old as reference)						
50–65	−1.564	0.583	−0.172	−2.683	0.008	−2.715–−0.413
>65	−2.334	0.642	−0.210	−3.635	<0.001	−3.599–−1.069
Education level (with junior high school and below as reference)						
High school or technical secondary school	1.243	0.556	0.163	2.236	0.026	0.148–2.338
Junior college or above	2.032	0.601	0.189	3.380	0.001	0.848–3.216
Comorbidities (with no as reference)	−1.693	0.547	−0.169	−3.096	0.002	−2.773–−0.613
BMI (with ≤23.9 as reference)						
24.0–27.9	−1.228	0.539	−0.136	−2.279	0.024	−2.291–−0.165
>27.9	−2.091	0.608	−0.172	−3.439	<0.001	−3.289–−0.893
Anxiety score (GAD-7, with no anxiety as reference)						
Mild anxiety	−0.832	0.469	−0.109	−1.774	0.078	−1.760–0.096
Moderate anxiety	−2.134	0.512	−0.199	−4.168	<0.001	−3.143–−1.125
Severe anxiety	−3.529	0.661	−0.269	−5.338	<0.001	−4.826–−2.232
Social support rating scale (SSRS, with low as reference)						
Middle	1.228	0.541	0.135	2.270	0.025	0.161–2.295
High	2.662	0.626	0.231	4.251	<0.001	1.423–3.901
General self-efficacy scale (GSES, with low as reference)						
Middle	0.682	0.472	0.074	1.445	0.150	−0.248–1.612
High	2.073	0.537	0.206	3.861	<0.001	1.010–3.136

*R*^2^ = 0.682, after adjustment, *R*^2^ = 0.668, *F* = 49.732, *P* < 0.001.

## 4 Discussion

This cross-sectional study systematically evaluated out-of-hospital fluid management among 184 patients with CHF. The overall score indicated a moderate level of self-management ability. Multiple linear regression analysis identified seven independent factors associated with this ability: better fluid management performance was significantly associated with younger age, higher educational level, stronger social support, and greater self-efficacy; conversely, poorer performance was linked to the presence of comorbidities, higher BMI, and higher levels of anxiety. This study provides insights into out-of-hospital fluid management for CHF patients, a crucial aspect of self-care that can significantly impact their long-term health outcomes. While this study may be of more direct relevance to primary care providers and those working in ambulatory settings, the findings can support hospital-based clinicians in understanding the broader psychosocial factors influencing patient behavior outside the hospital.

In this study, younger ages were associated with better fluid management ability. This may be attributed to generally higher digital health literacy and greater familiarity with technology-facilitated self-monitoring, which are more prevalent in younger populations and support consistent self-care practices (20). Additionally, younger patients often have a lower burden of comorbid conditions and better physical function, which can reduce the complexity of daily management (21). Higher educational level was also a significant predictor of better fluid management. Education is a well-established determinant of health literacy, which influences the ability to understand, remember, and act on complex medical instructions related to diet, medication, and symptom monitoring (22). Tailoring education to match patients' literacy levels is therefore crucial for equitable care. The presence of comorbidities was independently associated with poorer fluid management. Conditions such as hypertension, diabetes, or chronic kidney disease increase the overall illness burden, therapeutic complexity (e.g., polypharmacy), and the risk of conflicting dietary or fluid recommendations, making consistent self-care more challenging (21).

Our results revealed that higher BMI was significantly associated with poorer fluid management ability. This finding aligns with established physiological understanding, as obesity presents several challenges for fluid management in heart failure patients. Higher BMI is frequently associated with conditions such as chronic inflammation, insulin resistance, and increased neurohormonal activation, all of which can promote sodium and water retention (23, 24). Additionally, obese patients often experience decreased physical mobility, which may impair their ability to engage in recommended self-care behaviors such as regular weight monitoring and appropriate physical activity (25). The presence of excess adipose tissue can also complicate accurate weight monitoring and mask early signs of fluid retention (26). These physiological and practical challenges likely contribute to the observed difficulty in maintaining effective fluid management among patients with higher BMI values. Therefore, clinical interventions should pay particular attention to weight management strategies for obese CHF patients, incorporating nutritional guidance, tailored physical activity recommendations, and close monitoring to support their fluid management efforts. However, clinicians and patients should be aware that weight fluctuations can occur due to non-fluid related factors, and its utility as a sole indicator of fluid overload is limited, particularly in patients with obesity.

Although both anxiety and depressive symptoms were assessed, only anxiety demonstrated a significant independent association with fluid-management ability. This finding is consistent with prior literature suggesting that anxiety more strongly influences moment-to-moment self-care behaviors in heart-failure populations. Anxiety tends to heighten symptom vigilance, impair concentration, and disrupt daily routines, which may directly interfere with tasks such as weight monitoring, fluid restriction, and prompt response to symptom changes. In contrast, depressive symptoms did not remain significant after adjustment, likely because depression and anxiety share overlapping pathways, and anxiety often exerts the more immediate behavioral impact. Prior studies have similarly reported that anxiety-but not depression predicts poorer adherence and self-care execution when both are included in the same model (27), and anxiety predicts event-free survival mediated by nonadherence (28).

Our findings-that higher self-efficacy and stronger social support are associated with better out-of-hospital fluid management, and that anxiety, comorbidities, education and age influence self-management-are consistent with recent international research on heart-failure self-care. Current guideline and review evidence emphasize patient education, symptom monitoring (including weight/volume status), and psychosocial support as core elements of self-care. The 2022 AHA/ACC/HFSA guideline highlights the need for individualized education and support to improve adherence to self-care behaviors (9). Several observational studies report persistent gaps in self-care and identify similar determinants: lower social support and lower self-efficacy predict poorer self-care behaviors and worse outcomes, while anxiety and a higher comorbidity burden are linked to lower adherence (21). A 2024 review focused on fluid restriction and fluid-related self-management reiterates that practical skills (weight monitoring, adjusting fluid intake) and tailored counselling are essential to achieve effective fluid balance outside hospital (14). The qualitative and mixed-methods studies published in 2024 emphasize caregiver involvement and system-level barriers (access to education, technology, and follow-up), which help explain why education level and younger age were associated with better self-management in our sample (20).A study (29) focusing on Black individuals with heart failure in South Florida found that social support was a key facilitator for physical activity engagement. Meraz et al. identified that higher resilience and health literacy independently predicted better medication adherence, while greater perceived social support was the only significant predictor of improved heart-failure self-care after controlling for age and depressive symptoms (30). The study by Jennings et al. (22) found that more threatening illness perceptions and poorer health literacy were strongly and independently associated with higher levels of anxiety and depression, as well as poorer health-related quality of life in patients with coronary heart disease (22).

Based on our findings that self-efficacy and social support significantly are associated with better fluid management, healthcare providers should implement targeted strategies to enhance these factors in clinical practice. To improve self-efficacy, clinicians can employ motivational interviewing to strengthen patients' confidence, conduct skill-building workshops for practical self-management techniques, and establish peer support groups for shared learning experiences. For enhancing social support, healthcare teams should develop family-involved care plans that educate family members on providing appropriate supervision and reminders, utilize social workers to connect patients with community support resources, and implement mobile health technologies that enable family participation in monitoring and encouragement. These evidence-based approaches address both individual psychological factors and social environmental determinants, creating comprehensive support systems that can significantly improve CHF patients' fluid management capabilities and overall disease outcomes.

In the context of the Chinese healthcare system, our findings also suggest meaningful opportunities for community-based and digital self-management interventions. Nurse-led telemonitoring programs, community health-service station follow-up, and mobile health applications designed for chronic disease management have shown potential in enhancing daily symptom surveillance, fluid-status monitoring, and adherence to self-care behaviors. These models align with China's “Internet + Healthcare” initiatives and may be particularly beneficial for older adults, patients with limited health literacy, or those living in rural areas. Integrating these community-anchored and digital tools into routine follow-up could further strengthen out-of-hospital fluid-management capabilities among CHF patients.

While this study provides valuable insights into the self-reported fluid management practices of CHF patients, several limitations should be acknowledged. First, the use of a convenience sampling method from a single center, together with the relatively small sample size, may introduce selection bias and limit the representativeness of the sample, thereby affecting the generalizability of the findings to broader CHF populations. Second, our reliance on self-reported, questionnaire-based data collection-although appropriate for assessing patient-reported outcomes-may be subject to recall and social desirability bias and lacks correlation with objective clinical indicators of fluid status such as serial weight measurements, biomarkers (e.g., N-terminal pro-B-type natriuretic peptide), or bioimpedance data. This limitation prevents us from establishing direct associations between reported behaviors and physiological changes in fluid balance. Third, the cross-sectional nature of our study design restricts our ability to infer causal relationships or evaluate longitudinal changes in fluid management behaviors. Fourth, although we included important psychosocial variables such as anxiety and self-efficacy, other relevant factors (e.g., cognitive function, health literacy, or social determinants of health) were not assessed. Fifth, data on pharmacologic treatment, particularly the use and dosage of loop diuretics, a cornerstone of decongestive therapy-were not collected. This omission limits our ability to adjust for a key medical determinant of fluid status and to disentangle its effect from self-care behaviors. Sixth, potential variations in clinical management strategies and health education practices that may influence patient self-care behaviors were not controlled for. These limitations highlight important directions for future research. Prospective, multicenter, and longitudinal studies incorporating both self-report measures and objective digital monitoring tools (e.g., smart scales, wearable devices, or mobile health applications) are warranted to provide a more comprehensive and accurate assessment of fluid management in CHF patients and to enhance the generalizability and robustness of the findings.

## 5 Conclusions

Age, education, comorbidities, BMI, anxiety, social support, and self-efficacy significantly influenced out-of-hospital fluid management in CHF patients. Younger, better-educated patients with higher self-efficacy and social support performed better, whereas patients with more comorbidities, higher BMI, and higher anxiety had greater difficulty. Clinical management should integrate physiological, psychological, and social strategies, providing personalized education and support to enhance self-management and quality of life.

## Data Availability

The datasets presented in this study can be found in online repositories. The names of the repository/repositories and accession number(s) can be found in the article/Supplementary Material.
